# This is for you: Social modulations of proximal vs. distal space in collaborative interaction

**DOI:** 10.1038/s41598-019-51134-8

**Published:** 2019-10-18

**Authors:** Roberta Rocca, Mikkel Wallentin, Cordula Vesper, Kristian Tylén

**Affiliations:** 1 Department of Linguistics, Cognitive Science and Semiotics, Aarhus University, Aarhus, Denmark; 2 Interacting Minds Centre, Aarhus University, Aarhus, Denmark; 3 Center of Functionally Integrative Neuroscience, Aarhus University, Aarhus, Denmark

**Keywords:** Language, Social behaviour, Cooperation, Human behaviour

## Abstract

Human spatial representations are shaped by affordances for action offered by the environment. A prototypical example is the organization of space into *peripersonal* (within reach) and *extrapersonal* (outside reach) regions, mirrored by *proximal* (this/here) and *distal* (that/there) linguistic expressions. The peri-/extrapersonal distinction has been widely investigated in individual contexts, but little is known about how spatial representations are modulated by interaction with other people. Is near/far coding of space dynamically adapted to the position of a partner when space, objects, and action goals are shared? Over two preregistered experiments based on a novel interactive paradigm, we show that, in individual and social contexts involving no direct collaboration, linguistic coding of locations as proximal or distal depends on their distance from the speaker’s hand. In contrast, in the context of collaborative interactions involving turn-taking and role reversal, proximal space is shifted towards the partner, and linguistic coding of near space (‘this’ / ‘here’) is remapped onto the partner’s action space.

## Introduction

## Representing Space for Action and Interaction

A significant part of human spatial cognition is influenced by affordances for action and interaction offered by the environment^1,2^. The tendency to encode functional features of objects and locations finds expression in the organization of space into an immediate *peripersonal* and a distal *extrapersonal* region. This distinction maps onto a contrast between objects *within* and *outside* manual reach, optimizing sensorimotor representations for manual action and defensive behavior^3,4^.

The link between manual affordances and spatial cognition has been explored extensively in the literature^5,6^. However, the majority of studies on the topic have focused on solitary individuals manipulating an object, while a considerable portion of our everyday behaviors unfolds in the context of face-to-face social exchanges where multiple individuals engage in a dynamic interaction with each other and with the environment^7,8^.

In a range of situations, from passing the salt at the dinner table to building a new house, we find ourselves in situations where actions are performed together with other people and coordinated via language. In these cases, space is often shared between interlocutors, and objects in this space lend themselves to collaborative joint attention^9^ and action^10–12^. Within such joint attentional scenes, objects’ affordances for manipulation emerge as a result of complex interactions between their distance to other objects and agents, and their contribution to specific action goals. Representations of space are thus likely to be dynamically modulated by functional properties of objects with regards to both individual and social action possibilities.

These observations lead to a number of specific predictions. First, given the action-oriented nature of spatial representations, peripersonal space is hypothesized to be biased towards hand-centered coordinates, a format which facilitates fast movement execution^13^.

Second, in activities involving other agents, functional representations of space may undergo structural changes to encode object affordances for joint action^14,15^. Such dynamical social adaptations of near/far spatial coding are expected on the basis of a large number of studies on joint action showing that interlocutors spontaneously adapt to each other across a number of linguistic and non-linguistic behavioral measures, from the alignment of lexicon^16,17^ and situation models^18^, to subtle bodily sway^19^, and even heart rate^20^. Furthermore, studies on recipient design in language have shown that individuals flexibly adjust their utterances to the specific interlocutor and the overarching goal of the interaction^21,22^.

We tested these two hypotheses using a novel experimental paradigm that allows to investigate dynamic modulations of sensorimotor representations: (a) in a multi-person interactive context; (b) maintaining fundamental features of social task-oriented behavior; (c) in a controlled and low-dimensional space. Using spatial demonstratives (words like *this* and *that*) as an online linguistic index of representations of proximal space, we aimed at systematically investigating how egocentric action-oriented biases and context-specific social modulations interact in shaping representations of space in everyday social interactions.

### Demonstratives as linguistic indices of near/far spatial encoding

Spatial demonstratives are highly frequent lexical forms found across all natural languages^23^. Used in conjunction with pointing gestures and gaze cues, they constitute powerful interpersonal coordination devices^24,25^, that allow interlocutors to jointly attend to relevant locations, and to align on shared spatial representations. Whereas some languages have highly elaborate systems, most languages show a simple dyadic distinction between a so-called *proximal* (“this”, in English) and a *distal* (“that”) demonstrative^26^.

Experimental evidence shows that the contrast between these two forms is a reliable proxy of object reachability in individual contexts. Over a series of studies, Coventry and colleagues have shown that participants systematically prefer proximal demonstratives for objects within reach, and distal demonstratives for objects outside reach^27–29^. Moreover, the choice of demonstrative forms is sensitive to the same dynamic manipulations affecting the boundaries of peripersonal space^30^. For instance, participants spontaneously extend the space for which they use a proximal demonstrative if they point to referents using a stick^31,32^, and are similarly affected by perceptual and psychological parameters of the referent, such as its visibility, ownership and familiarity, when choosing a demonstrative form^27^.

### The present study

Over two experiments, we investigated how coding of objects and locations as *near* vs. *far* is influenced by affordances for manual action, and modulated across contexts of social interaction. Using a novel experimental paradigm, we presented participants with targets appearing on a two-dimensional horizontal plane and asked them to point and refer to them using demonstratives. In Experiment 1, participants performed the task alone or with a confederate, who was either engaged in a complementary naming task or in a collaborative communicative task. We hypothesized that targets located closer to the speaker on the sagittal plane and rightwards on the lateral plane (all participants were right-handed) would be more likely to be identified as proximal, which would support the hypothesis that proximal/distal encoding is tied to manual affordances of the referent. Crucially, we hypothesized that, in the context of collaborative interaction, targets located closer to the partner (that is to the participant’s left) would be more likely to be labelled as proximal than in individual or non-collaborative social contexts, indicating that in this condition manual biases are modulated by social affordances. Results from Experiment 1 have been previously reported in conference proceedings^33^. In the present paper, these results are discussed more extensively and complemented with a second experiment, building on the interactive components of the social manipulation. In Experiment 2, we used a similar setup, but enhanced the interactive aspects of the task by introducing turn-taking and role reversal. Both experiments were pre-registered on the Open Science Framework. Pre-registrations, data and code are publicly available at osf.io/qjxg9/.

## Experiment 1

### Methods

#### Participants

Eighty right-handed participants (female = 43, age range = 19–48, median = 26, sd = 7.6) with Danish as first language took part in the experiment in return for monetary compensation. All participants gave written informed consent. The study received ethical approval from the Human Subjects Committee of the Cognition and Behaviour Lab at Aarhus University, and it was carried out in accordance with local ethical guidelines and procedures.

#### Design and procedure

Upon arrival to the lab, participants were instructed that they would be presented with a spatial working memory test, and that they were assigned to the “linguistic condition”. However, unbeknownst to the participants, there was no between-participant manipulation, that is, all participants did the same version of the experiment (in line with^27–29^).

Participants stood by the inferior edge of a 40” screen, placed horizontally on a table. At each trial, a grid of circles would appear on the screen. After 500 ms, the grid disappeared and two target shapes (circles, triangles, squares, hexagons, stars) appeared on the screen for a random interval between 200 ms and 800 ms. The position of targets was randomized across trials. The grid would then reappear and the participant was prompted to designate the target positions. Participants were instructed to remember the locations of targets, then point to them while referring to the locations with the Danish demonstratives *den her* or *den der*, equivalent to the English *this* and *that*. Participants were explicitly instructed to use both demonstrative forms at each trial, one for each target, and were reminded to do so whenever they used only one of the expressions. No explicit instructions were given on the order of the pointing nor on the order of deictic forms. There were 132 trials per condition per participant.

Participants performed the task across three conditions. In the *baseline condition*, participants performed the task alone. In the *complementary condition*, a confederate stood to the left of the participant and named the target shapes (e.g. *star*, *circle*) after the participant had finished pointing. In the complementary condition, the two tasks were mutually independent. Neither the participant nor the confederate relied on the information provided by the other person in order to be able to perform their own task. In contrast, in the *collaborative condition*, the confederate’s task depended on the information conveyed by the participant’s pointing. In this condition, the confederate closed his or her eyes during target exposure and only opened them after hearing a click sound signifying the re-appearance of the grid. The participant then pointed at the location of both targets and referred to them using demonstratives. This allowed the confederate, who did not have perceptual access to the targets, to report their positions on a touch screen device placed next to the screen.

**Figure 1:**
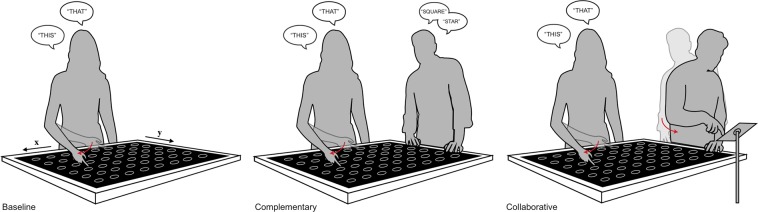
Experimental setup for the three conditions.

The authors and two student assistants took turns in the role of experimenter (live-coding the participants’ responses) and confederate. The baseline was always performed first. The order of the complementary and collaborative conditions was counterbalanced across participants. The setup of the experiment is displayed in Fig. 1.

Responses were coded as “invalid”, if the participants reported not having seen where the targets had appeared, when participants failed to use both demonstrative forms, or when the experimenter could not hear the participant’s response. The experiment was not videotaped, and no offline coding of responses was performed.

There was a total of 286 invalid trials out of 31680 total trials. Missing trials were distributed as follows: 76 in the *baseline* condition (0.95 per participant on average), 77 in the *complementary* condition (0.96 per participant on average), 133 in the *collaborative* condition (1.66 per participant on average). Invalid trials were excluded from the analysis.

At the end of the experiment, participants were asked to report what they thought the experiment was about, then debriefed. None of the participants reported to have realized that their use of demonstratives was the behavior of interest and they reported no awareness of how they had distributed the demonstrative forms spatially.

#### Analysis

**Figure 2:**
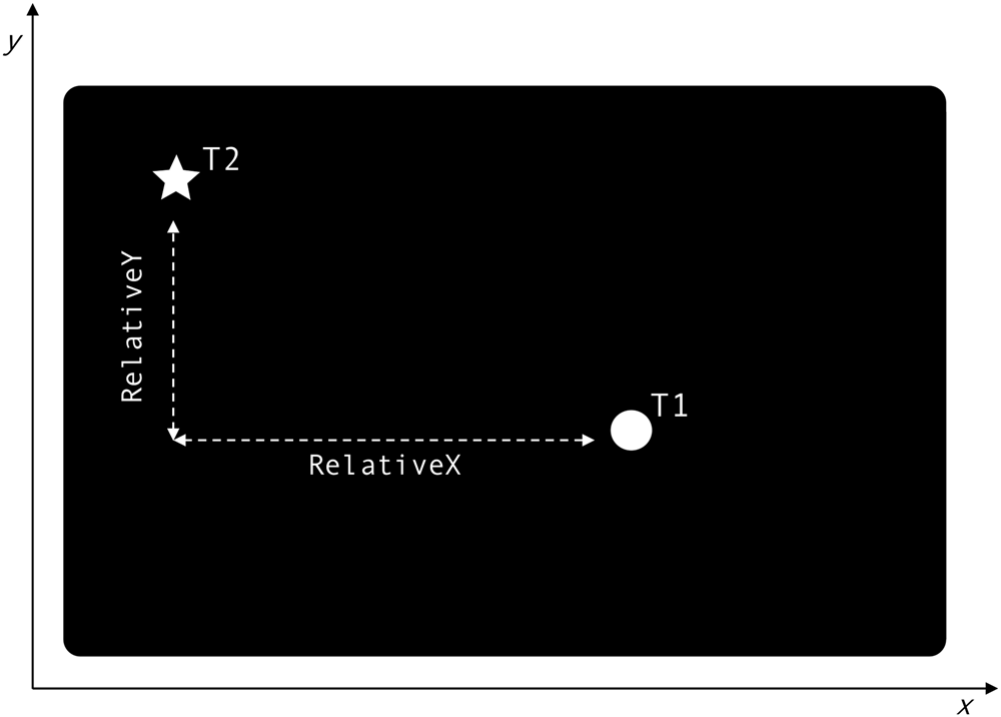
Computation of relative distances between T1 and T2 on the sagittal (RelativeY) and lateral axis (RelativeX). The coordinate system is oriented from left to right for the x axis, and from the bottom to the top of the screen for the y axis. RelativeX is computed by subtracting the x coordinate of T2 from the x coordinate of T1. Since the x coordinate of T1 is larger, in this example RelativeX takes positive value. RelativeY is computed by subtracting the y coordinate of T2 from the y coordinate of T1. Since in this example the y coordinate of T1 is smaller, RelativeY takes a positive value.

The relative distances between the x coordinates and the y coordinates of the two targets were used as predictors for a mixed effects logistic regression, fitted using the *glmer* function from *lme4* package in RStudio^34^. For each trial, one of the two targets (henceforth: T1) was randomly selected and logged as target of interest. Relative distances on each of the axes were computed by subtracting the x coordinates and the y coordinate of the competitor target (henceforth: T2) from those of T1. The relative distance on the x axis (*RelativeX*) took positive values if T1 was further to the right than T2, whereas their distance on the y axis (*RelativeY*) took positive values if T1 was further away from the speaker than T2 on a sagittal axis. Figure 2 exemplifies how relative distances were computed in a sample trial.

Notice that relative distances combine information on distance between T1 and T2, and distance between T1 and the speaker. To provide a concrete example, large positive values of relative distance on the y axis not only indicate larger distance between the targets, but they also indicate that T1 is further away from the speaker, while the opposite holds for negative values. This metric makes it possible to investigate how the likelihood of coding a referent as *proximal* or *distal* is influenced by its position relative to the speaker and to competing referents, both relevant factors in linguistic organization of space into proximal vs. distal locations^28,35^.

The *fixed effects* structure of the model included the relative distance between the two targets on the y axis (henceforth: *RelativeY*), the relative distance between the two targets on the x axis (henceforth: *RelativeX*), and a categorical predictor for condition (henceforth: *Condition*), as well as all interactions. The demonstrative word chosen to refer to T1 was used as outcome variable in the model. The distal demonstrative (*that*) was set as reference level, while the proximal form (*this*) was coded as success outcome.

The *random effect* structure included random intercepts for each participant as well as random slopes for RelativeY. The decision to only include slopes for RelativeY was made during the preregistration phase. The rationale was to keep the random effects structure simple enough to avoid potential convergence issues and therefore post-hoc adaptations of the model, while still accounting for between-participant variability in sensitivity to the expectedly most prominent effect.

Parameters were estimated using maximum likelihood estimation with Laplace approximation. The power simulation for the model is reported in the preregistration, yielding 70–100% power for fixed effects and two-way interactions. Planned contrasts for the categorical predictor *Condition* compared the participant’s behavior in the baseline condition with cumulative behavior in the social conditions, as well as the complementary condition against the collaborative condition.

#### Expected effects

On the basis of the hypotheses outlined above, we expected a negative main effect of *RelativeY*. More specifically, we hypothesized that the further away from the speaker T1 was on the sagittal plane, the less likely this target would be labelled as “proximal”.

Additionally, we expected a positive main effect of *RelativeX*. This effect would indicate that the more to the speaker’s right T1 was relative to T2, the more likely T1 would be labelled as “proximal”.

These effects would support the hypothesis that proximal/distal encoding is linked to manual affordances of the referent. Additionally, we expected *RelativeX* to interact with *Condition*. This indicates that the right-ward bias expressed by the effect of *RelativeX* would be significantly less pronounced in the collaborative conditions. In this condition, we expected targets located closer to the partner – that is, more to the participant’s left – to be relatively more likely to be labelled as proximal than in individual or non-collaborative social contexts. Given the binary nature of the outcome (proximal vs. distal), this prediction can equivalently be expressed in terms of right-ward targets being more likely to be labelled as distal in the collaborative condition, compared to individual or non-collaborative social contexts.

Detecting this effect would support the hypothesis that, in contexts of collaborative interaction, manual biases are modulated by social affordances.

## Results

### Main effects of distance on the sagittal and lateral axes

**Figure 3:**
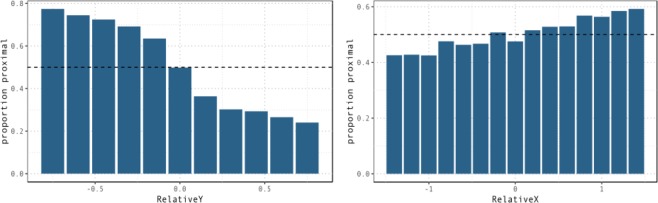
Proportion of proximal demonstratives used to denote T1 as a function of its distance from the competing target (T2) on the *sagittal* (left) and *lateral* (right) axis across all conditions. As T1 moves further away from the participant on the sagittal axis, that is as the value of *RelativeY* increases, the probability of participants coding it as *proximal* decreases. As T1 moves further to the participant’s right, that is as the value of *RelativeX* increases, the probability of participants coding it as *proximal* increases, speaking in favour of a right-lateralized bias for proximal space.

Figure 3 displays the proportion of proximal demonstratives as a function of *RelativeY* and *RelativeX* across conditions.

The proportion of proximal demonstratives decreases as RelativeY increases, i.e. as T1 moves further away from the speaker on the sagittal axis relative to T2. Additionally, Fig. 3 displays an increase in proportion of proximal demonstratives as a function of an increase in the value of *RelativeX*, i.e. as T1 moves further to the participant’s right.

The mixed effects logistic regression model with *RelativeX*, *RelativeY* and *Condition* as predictors, and including all interactions, confirms the statistical reliability of these patterns. The model displays a significant effect of *RelativeY*, β = −2.59, se = 0.27, z = −9.69, p < 0.001 and of *RelativeX*, β = 0.32, se = 0.02, z = 16.78, p < 0.001.

Taken together, the effects of relative distance of targets on the sagittal and lateral plane are in line with the idea that proximal space is intrinsically linked to manual affordances. As distance from the pointing hand decreases, objects become more likely to be identified as *proximal*.

### Effect of social manipulations

Planned contrasts reveal a significant interaction between *RelativeX* and *Condition* when comparing the *complementary* and *collaborative* condition, β = 0.05, se = 0.02, z = 2.17, p = 0.03. No such effect is observed when cumulatively comparing the baseline to the social conditions, β = −0.001, se = 0.01, *z* = − 0.08, *p* = 0.936.

The interaction between *RelativeY* and *Condition* reaches statistical significance both in the contrast between the baseline and the two social conditions, β = −0.07, se = 0.03, z = −2.51, p = 0.012, and between the *complementary* and *collaborative* condition, β = −0.11, se = 0.05, z = − 2.45, p = 0.014. None of the three-way interactions reached statistical significance.

**Figure 4:**
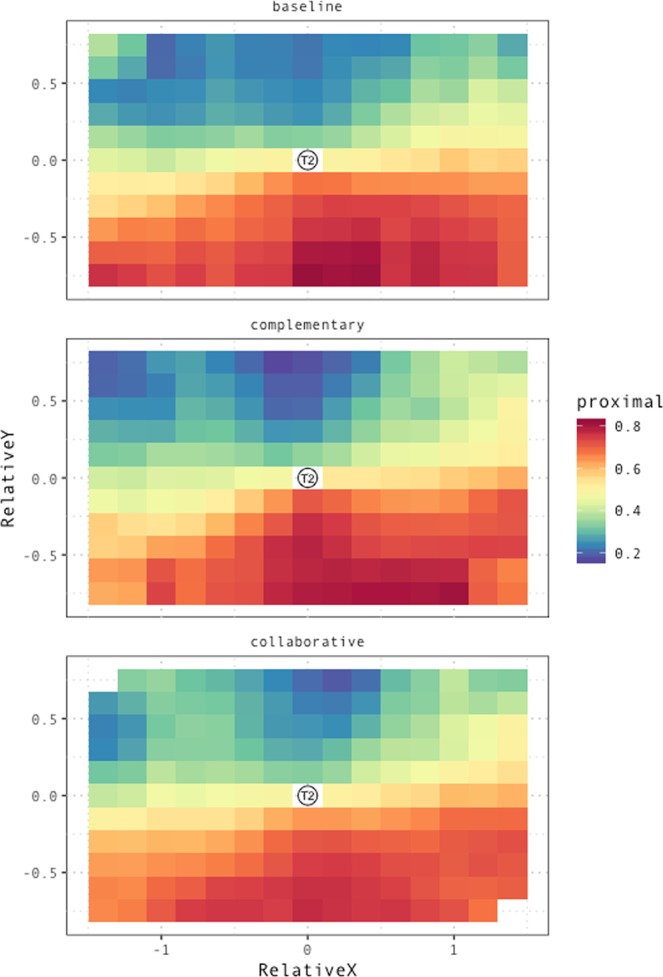
Proportion of trials in which T1 is coded as *proximal* as a function of distance of T1 from T2 on the sagittal (*RelativeY*) and lateral (*RelativeX*,) axis. Larger *RelativeY* values indicate that T1 is further away from T2 (and from the participant) on the sagittal axis. Larger *RelativeX* values indicated that T1 is further to the right of T2. The center of the plot represents the position of T2, providing a reference for the interpretation of relative distance values on the x and y axis.

Figure 4 displays the observed proportion of proximal outcomes as a function of both *RelativeX* and *RelativeY* across conditions, providing an overview of the interplay between proximal/distal coding of space and the three experimental variables included in the statistical analysis.

The heatmaps clearly display the effect of *RelativeY* across all conditions. The right-lateralized bias on the x axis detected in the statistical analysis is particularly salient in the baseline and complementary condition, whereas it is slightly attenuated in the collaborative condition. Maps are smoothed by averaging across 8 nearest neighbors in x-y 2D space.

**Figure 5:**
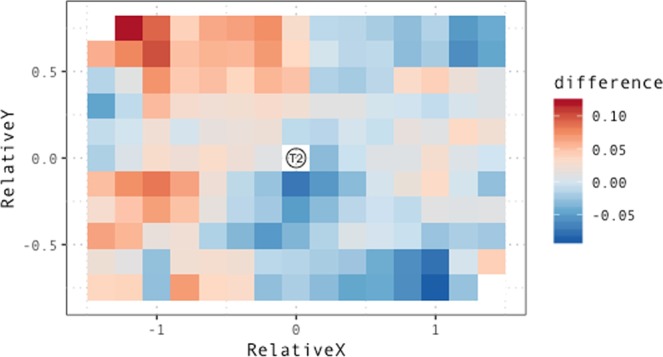
Difference in proportion of proximal demonstratives between the collaborative and complementary condition. Positive values (red) indicate higher proportion of proximal demonstratives in the collaborative condition, while negative values denote higher proportion of proximal demonstratives in the complementary condition. In the collaborative condition, participants code targets located towards their right as distal more often than in the complementary condition. Conversely, participant have a stronger tendency to code as *proximal* those targets that are located towards the left side of the screen, an index of representations of proximal space shifting towards the interaction partner. The difference is especially salient on the top-left of the plot, indicating that participants display a markedly higher tendency for participants to use proximal demonstratives for targets that are located towards the left even when they are further away from them on the sagittal axis. This suggests that near/far coding of spatial locations in collaborative contexts might depend to a higher extent on distance from the partner, rather than on distance from oneself.

Figure 5 zooms in on the differences between the collaborative and the complementary condition, displaying the difference in proportion of proximal demonstratives for all combinations of values of *RelativeX* and *RelativeY*.

A full overview of the estimates from the statistical model is provided in Supplementary Table S1.

### Interim discussion

In Experiment 1, we detected a functional bias in favor of the pointing hand when participants performed the task individually. The hand-oriented bias persisted if two participants were performing independent tasks. However, in contexts of collaborative interaction, that is, where participants depended on each other to perform their tasks, proximal space is shifted towards the partner. Interestingly, we observed social modulations of proximal/distal coding of space both in terms of expected left-ward modulations in proximal space and in terms of an unpredicted interaction between condition and relative distance on the sagittal axis (RelativeY). These modulations on the sagittal axis indicate that, while sagittal distance played a major role in speakers’ proximal/distal coding of space when participants performed the task individually or with a confederate performing an independent task, the effect of this factor was significantly less pronounced in contexts of collaborative interaction. This might in turn indicate that, in collaborative contexts, speakers drift away from coding locations as proximal vs. distal uniquely based on distance from their own body, towards a coding strategy that takes (also) the partner’s body as reference. Involvement in a collaborative interaction might not simply influence coding of proximal vs. distal space in the form of modulations of individual lateralized biases, but rather trigger a deeper change in the very reference frame against which proximity is evaluated.

In Experiment 2 we aimed at replicating both the expected and unexpected findings from Experiment 1 adding one more naturalistic component to the social tasks. In Experiment 2, in fact, participants take turns in performing speaker and addressee tasks introduced in Experiment 1. This role alternation mirrors the turn-taking structure of natural linguistic interaction, and enhances the collaborative nature and ecological validity of the task.

## Experiment 2

### Methods

#### Participants

We collected data from 40 pairs of participants (80 individual participants, female = 51, age range = 18–36, median = 22, sd = 2.68). All participants were right-handed and native speakers of Danish. Participants within each pair were recruited independently, and reported not knowing each other in advance. All participants gave written informed consent. The study received ethical approval from the Human Subjects Committee of the Cognition and Behaviour Lab at Aarhus University, and it was carried out in accordance with local ethical guidelines and procedures.

#### Design and rationale

Participants took part in the experiment in pairs. The experiment was performed using the same horizontally placed 40” screen as in Experiment 1. One participant (P1) stood by the inferior edge of the screen. The other participant (P2) stood by the adjacent side of the screen to the left of P1, in the same position occupied by the confederate in Experiment 1. In this experiment, participants performed the same pointing and naming tasks as in Experiment 1 over two conditions, a *complementary* and a *collaborative* condition. In both conditions, at each turn, one of the participants had to point at targets, while the other had to name their shape. Differently from Experiment 1, however, the two participants alternated in performing each of the two tasks, switching roles at every trial. Introducing a turn-taking structure allowed the task to better mirror the symmetrical structure of naturalistic linguistic interaction.

As clarified in detail below, the complementary and collaborative conditions differed in whether the partner performing the naming task had perceptual access to the targets lighting up on screen. In the complementary condition, he/she was able to see which targets lit up. In the collaborative condition, the participant performing the naming task was not able to *see* which targets lit up, thus *depending* on the information conveyed by the partner’s pointing to perform his/her own task. This mirrors the characteristics of the distinction between complementary and collaborative condition from Experiment 1, but it further improves the design in terms of experimental control, since two social conditions involving the *same* task are now compared.

The presence of a co-participant instead of a confederate also aimed at better mirroring the characteristics of spontaneous real-life interactions, and enabled us to analyse the linguistic behavior of two interacting participants, rather than only coding for the behavior of one individual at a time.

#### Procedure

At the beginning of each trial, a 7 × 7 square grid of different shapes (triangles, squares, circles, stars and hexagons) was displayed on screen for 500 ms. The distribution of individual shapes over the grid was randomized. All shapes were light grey against a black background. The square grid ensured that the extension of the target space along the sagittal and lateral axes was the same for both participants.

At each trial, two figures from the grid changed colour for a short interval (200–800 ms), to either red or green, trimmed to the same relative light and saturation as the default grey colour. In the *complementary* condition, P1 was instructed to respond to green targets, while P2 responded to red targets. After a click sound, depending on the colour of the figure, either P1 or P2 indicated which two shapes on the grid had lit up by pointing at their location and uttering a proximal and a distal demonstrative, as in Experiment 1. There were no specific constraints on the order in which demonstratives were to be used.

The other participant then named the *shape* of the figures that changed colour (“star”, “triangle” etc). Participants took turns in performing each task, that is, pointing to and naming the shape of targets. Both participants had visual access to the colour changes of the targets, thus to all the perceptual information necessary for their own task. The two tasks were therefore mutually independent.

In the *collaborative* condition, participants wore glasses with either red or green lenses. This prevented participants from being able to see targets of the same colour as their lenses. Trials unfolded as in the complementary condition. Participants were made aware that, at each trial, only one of them would be able to see the targets. In this condition, the participant who could see the targets changing colour indicated them using demonstrative forms and a pointing gesture. However, here the partner depended on this information to perform his/her task, that is, to name the figure shapes. In other words, in contrast to the *complementary* condition, here the demonstrative task was truly informative for the addressee’s naming task.

The order of presentation of conditions was counterbalanced across participant pairs. After completion of the experiment followed a short debriefing session.

The experiment was originally intended to include 170 trials per condition per pair, but the number of trials was readjusted over the course of the experiment due to lab time constraints. Overall, out of 40 pairs, 2 pairs performed the experiment with 170 trials per condition, 13 pairs performed 150 trials, and the remaining 25 pairs performed 160 trials.

As in Experiment 1, an experimenter live-coded participants’ responses. Responses could be coded as missing, when the participants reported not to be able to indicate the position of the targets, when participants failed to use both demonstrative forms, or when the participant’s response was unintelligible to the experimenter.

Overall, for P1, there were 225 trials with missing responses out of 3145 trials in the complementary condition, corresponding to 5.62 trials per participant on average, and 128, corresponding to 3.2 per participant on average, in the collaborative condition. For P2, there were 64 missing trials (1.6 on average) in the complementary condition, and 196 missing trials (4.9 on average) in the collaborative condition. In the post-experiment debrief, none of the participants reported having realized that demonstrative use was the behavior of interest. None of the participants reported awareness of the spatial distribution of his/her demonstrative use and how this was modulated across conditions.

**Figure 6:**
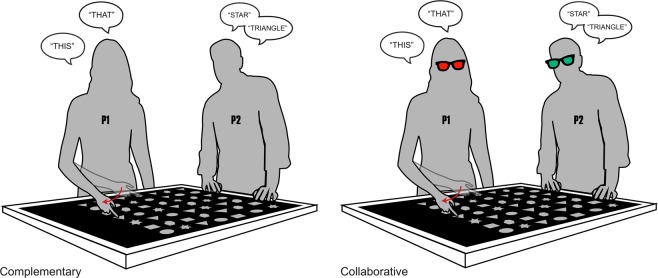
Setup for Experiment 2.

The experimental setup is represented in Fig. 6.

#### Analysis

We analysed the data using logistic mixed-effects regression as in Experiment 1. After discarding invalid responses, a total of 11967 out of 12580 data points were included in the analysis.

As in Experiment 1, at each trial, one target was randomly selected as reference target (T1), and the demonstrative used to refer to this target was used as outcome variable. Responses were coded as *distal* when participants used the distal forms *den der* when pointing to T1, and as *proximal* when participants used the proximal forms *den her* when pointing to T1. Distal was chosen as reference level, while proximal was the success outcome.

Distance between the two targets (relative to the position of each participant) was used as outcome variable. For trials where P1 responded, the relative distance on the x axis (*RelativeX*) was computed as the difference between the x coordinate of T1 and the x coordinate of T2. For this participant, relative distance on the y axis (*RelativeY*) was computed as the difference between the y coordinate of T1 and the y coordinate for T2. For P2, *RelativeX* was computed as the opposite of the difference between the y coordinate of T1 and the y coordinate of T2, while *RelativeY* was computed as the difference between the x coordinate of T1 and the x coordinate of T2. For both participants, *RelativeX* assumed positive values when T1 was located more towards the right of the participant, while *RelativeY* assumed positive values when T1 was located further away from the participant on the sagittal axis.

The complementary condition was set as reference level for the condition predictor variable. The categorical variable *color* coded for the color of the targets (*green* vs *red*), and therefore for which participants performed the demonstrative task at a specific trial. *Green* was set as reference level for this variable.

The fixed effects structure of the mixed effects logistic regression model included *RelativeX RelativeY*, *Condition* and *Color* as regressors, as well as the full interaction structure. The demonstrative form chosen to refer to T1 was used as outcome variable in the model. The random effects structure included a random intercept for each pair and random slopes for the effect of *RelativeY*. The model mirrors the one used for Experiment 1, with the addition of the *Color* regressor accounting for differences in behavior between the two participants.

Importantly, as participants are placed side by side, predictions on social modulations along the lateral axis have opposite directionality for P1 and P2. For P1, predictions were analogous to Experiment 1. The position of P1 coincides, in fact, with the position of the participant in Experiment 1. For this participant, the interaction partner is standing by his/her left. We therefore expected a left-ward shift in proximal space for this participant. This prediction translates into a negative two-way interaction between *RelativeX* and *Condition*.

However, predictions for modulations of *RelativeX* go in the opposite direction for P2. For P2, the interaction partner is placed to his/her right. A partner-oriented modulation along the lateral axis would therefore amount to an increased *right-ward* lateralized bias. This prediction translates into a (positive) three-way interaction between *RelativeX*, *Condition* and *Color*, showing that the way in which experimental conditions modulate the influence of *RelativeX* on proximal/distal coding of space has opposite directionality across the two participants.

As observed in Experiment 1, the effect of relative distance from one’s body along the sagittal axis tends to be reduced in the collaborative condition, arguably signaling a shift from a purely egocentric encoding of proximity, to a strategy in which the proximal/distal contrast accounts for distance from the interaction *partner*. On the basis of results from Experiment 1, we thus also expected a positive two-way interaction between *RelativeY* and *Condition*.

## Results

### Main effect of distance on sagittal and lateral axes

**Figure 7:**
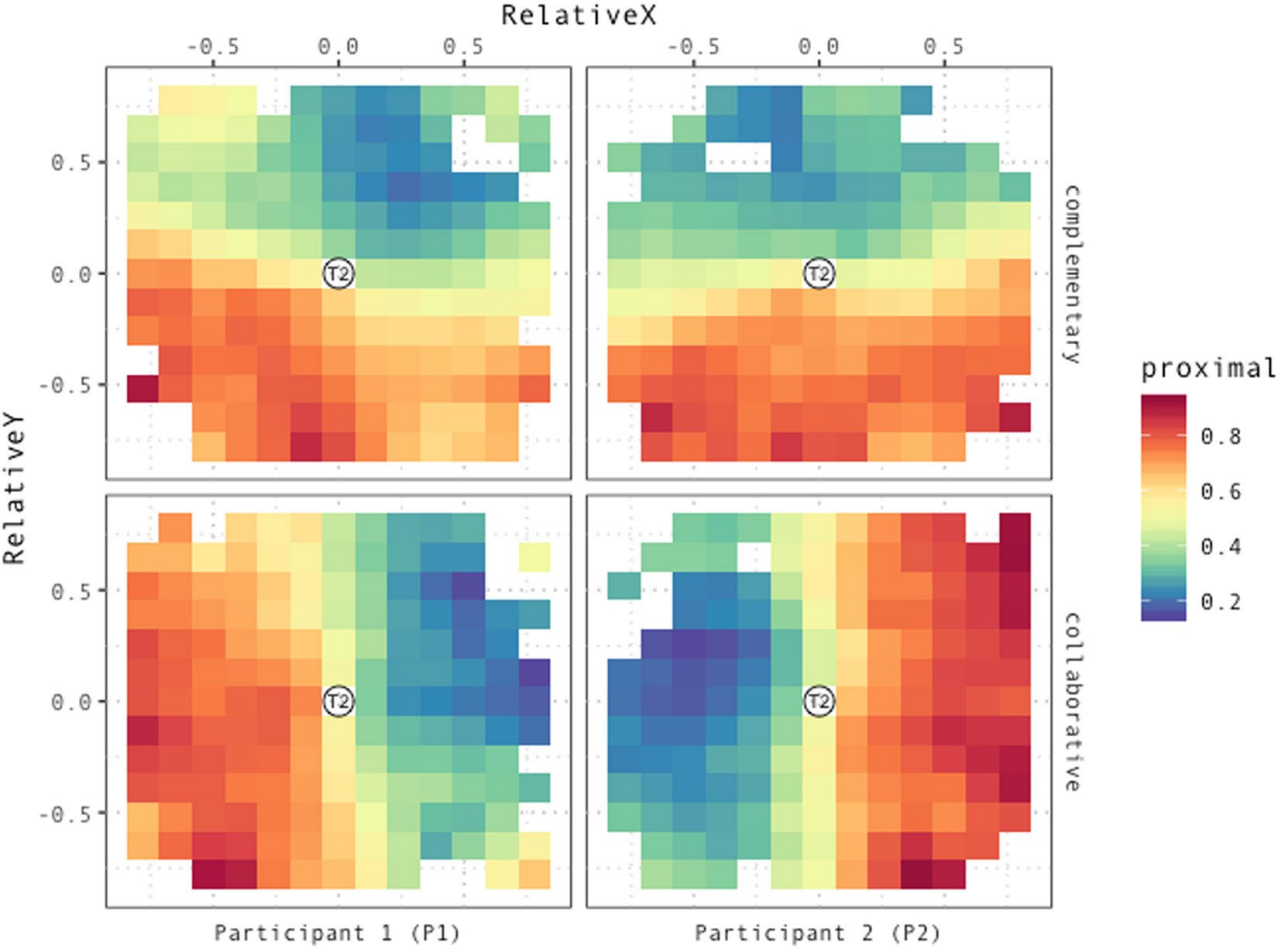
Proportion of trials in which T1 is coded as *proximal* as a function of RelativeX, RelativeY and Condition, for both P1 (the participant standing by the lower edge of the screen) and P2 (standing by the adjacent edge of the screen, to P1’s left). Colors code for the proportion of trials in which T1 is coded as *proximal*. Higher values of *RelativeX* correspond to T1 being located more towards the right of the participant relative to T2, whereas higher values of *RelativeY* correspond to T1 being located further away along the participant’s sagittal axis. Heatmaps in the top row display data for the complementary condition for both P1 (left panel) and P2 (right panel). Heatmaps in the bottom row display data for the collaborative condition for both P1 (left panel) and P2 (right panel).

Figure 7 displays the average proportion of proximal demonstratives as a function of relative distance on the participant’s sagittal and lateral plane, condition, and participants within pairs.

In the complementary condition (top row), for both participants, the probability of coding a referent as *proximal* highly depends on its distance from the competing referent on the participant’s *sagittal* axis (additionally, a minor left-lateralized bias towards the co-participant might already be in place for P1). Crucially, in the collaborative condition, participants remap proximal space onto the partner’s action space. As displayed by the heatmaps in the bottom row, in this condition both participants tend to label as *proximal* those locations which lie closer to their partner, i.e. towards their own left (P1), or right (P2). Maps are smoothed by averaging across 8 nearest neighbors in x-y 2D space.

The analysis displayed a main effect of *RelativeY*, β = −2.21, se = 0.23, z = −9.69, p < 0.001, denoting a decrease in proportion of proximal demonstratives as T1 moves further away from participants on the sagittal axis. This effect is in line with what was observed in Experiment 1.

Interestingly, however, the model also detects a main effect of *RelativeX*, β = −0.94, se = 0.12, z = −8.16, p < 0.001, indicating that the proportion of proximal demonstratives decreases as T1 moves further towards the *left* of the speaker relative to T2. This effect is driven by the behavior of P1. In fact, the interaction between *Color* and *RelativeX* indicates that no such bias (or a significantly smaller one) is there for P2, i.e. the participant standing by the lateral edge of the screen, β = 1.16, se = 0.16, z = 7.27, p < 0.001, who rather displays a small right-ward bias. Contrary to what was observed in Experiment 1, here proximal space might already be slightly shifted towards the addressee in the complementary condition.

### Effect of social manipulation

A significant interaction between *RelativeY* and *Condition* indicates that the effect of *RelativeY* is attenuated in the collaborative condition, β = 1.43, se = 0.17, z = 8.36, p < 0.001, and a three-way interaction with *Color* shows that this modulation is even stronger for P2 than for P1, β = 1.05, se = 0.24, z = 4.29, p < 0.001. This indicates that, overall, the probability of participants coding a target as *proximal* depends significantly less on the distance from the target on their sagittal axis (even less so for P2) when participants are collaborating.

**Figure 8:**
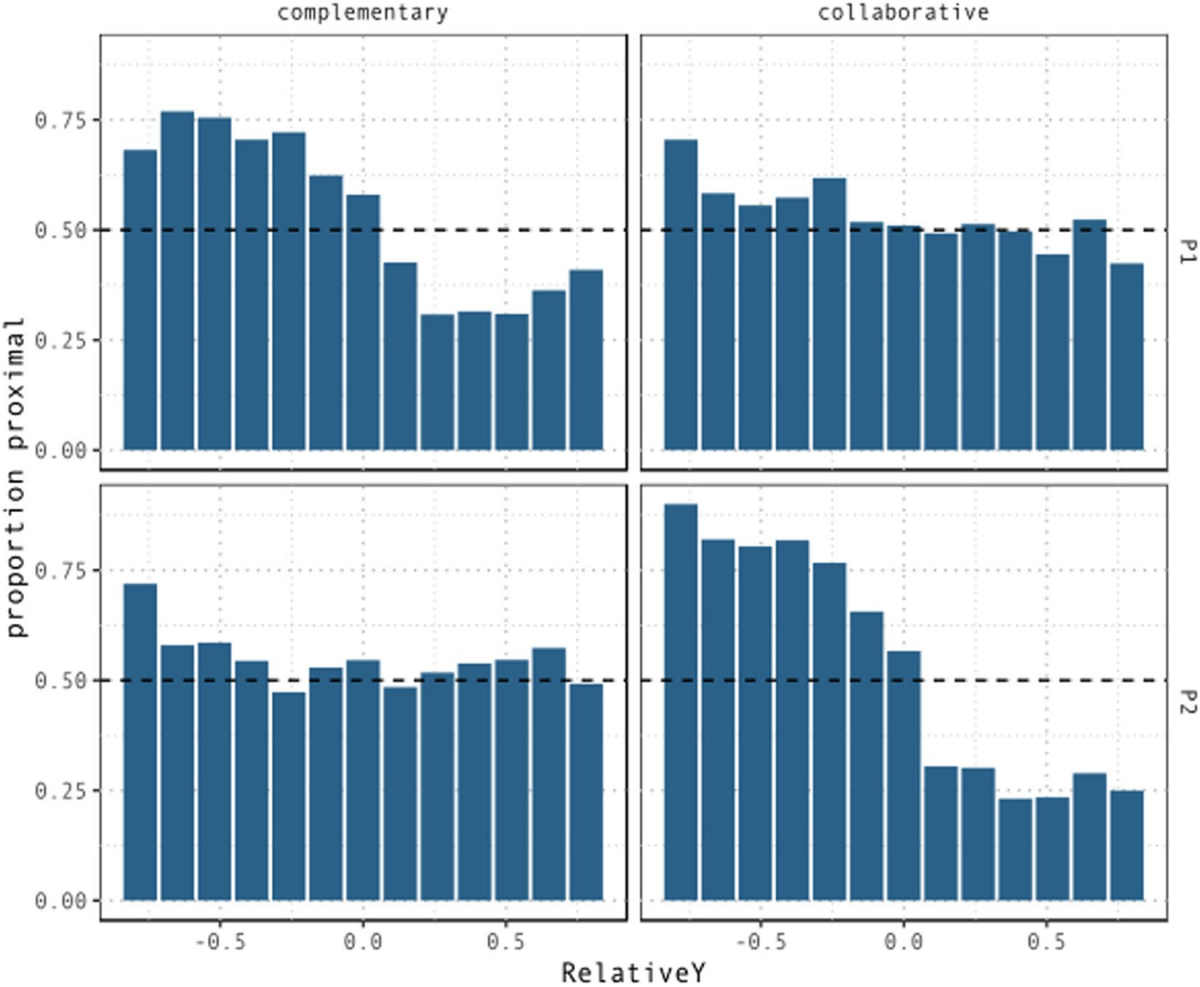
Proportion of trials in which T1 is coded as *proximal* by relative distance from T2 on the participant’s sagittal axis. In the complementary condition, the probability of participants coding a target as proximal decreases as it moves away from the speakers on the sagittal axis relative to competing targets. This effect is absent or significantly attenuated in the collaborative condition.

Figure 8 shows the proportion of proximal demonstratives across values of *RelativeY*, *Condition*, and participants within pairs.

Crucially, the effect of *RelativeX* is also modulated by *Condition*. In the collaborative condition, the tendency to code leftward locations as *proximal* was significantly stronger, an effect which is driven by P1’s behaviour, β = −1.8, se = 0.17, z = −10.43, p < 0.001.

**Figure 9:**
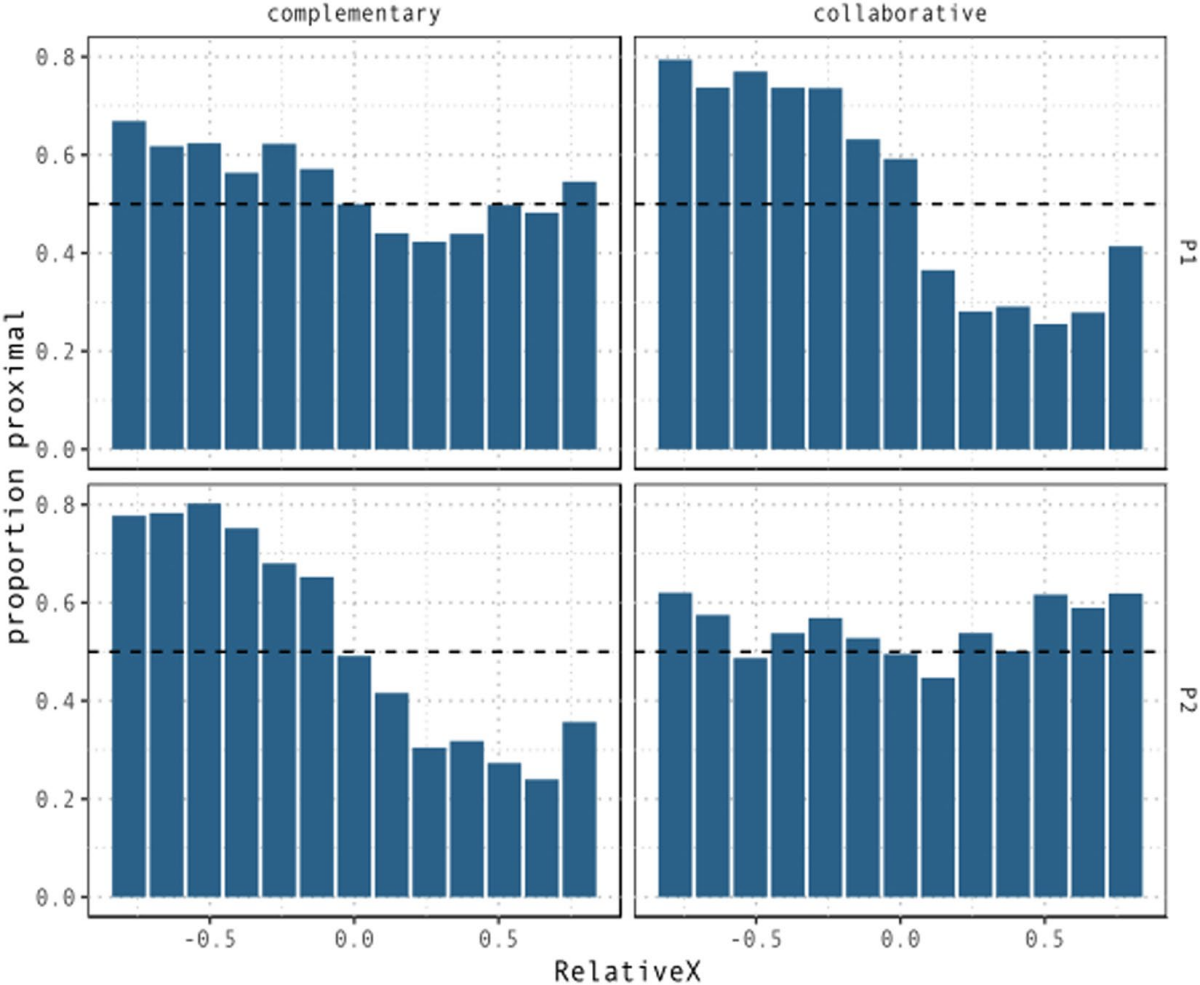
Proportion of trials in which T1 is coded as *proximal* by relative distance from T2 on the participant’s lateral axis. In the complementary condition, there seems to be a small bias towards labelling as proximal those targets located towards the partner on the lateral axis. This is observed in terms of a left-ward bias for P1, corresponding to higher proportion of proximal demonstratives for negative values of *RelativeX*, and the opposite right-ward bias for P2, corresponding to higher proportion of proximal demonstratives for positive values of *RelativeX*. In the collaborative condition, the effect of *RelativeX* is very strong. As targets move further to the left of P1, the probability of it being coded as *proximal* increases. The opposite effect is observed for P2. In this condition, *proximal* space is remapped as a function of the partner’s position.

In the collaborative condition, while distance on the sagittal plane becomes less relevant for both participants, the probability of coding a target as *proximal* now depends significantly more on the location of the target on the left-right axis. For P1, targets located towards the left, and therefore closer to the partner, are more likely to be labelled as proximal. A modulation in the opposite direction is observed for P2, who displays a significant tendency to code as proximal those targets that appear towards the right, i.e. towards the partner. This is indicated by the three-way interaction between *RelativeX*, *Condition*, and *Color*, β = 4.83, se = 0.25, z = 19.47, p < 0.001. These effects suggest that in the collaborative condition proximal space is shifted towards the partner for both participants. Figure 9 displays the proportion of proximal demonstratives across values of *RelativeX*, conditions and participants within pairs.

The model also displayed a significant interaction between *Color* and *RelativeY*, β = −0.4, se = 0.18, z = −2.26, p = 0.024, indicating that the effect of *RelativeY* was stronger for P2 than for P1.

A full overview of the estimates from the statistical model is provided in Supplementary Table S2.

### Exploratory analysis

The analyses reported above show that proximal space tends to be shifted towards the partner in collaborative contexts, arguably as a means to facilitate coordination for task performance. But what is the extent of such adaptations, and what type of coordination dynamics do they support? Do participants stably align on a new shared “compromise” reference frame? Or does each speaker fully remap *proximal* space onto the addressee’s action space, with participants oscillating between two distinct reference frames, each centred on the current addressee on a trial-by-trial basis?

If participants do converge on a single shared reference frame when collaborating, participants’ coding of space into *proximal* and *distal* locations in the collaborative condition should be more similar to their partner’s behaviour in the same condition, than to the partner’s behaviour in the complementary condition (where proximal/distal coding of space depends on one’s individual action space). On the contrary, if targets are coded as proximal or distal depending on their distance from the action partner, the opposite scenario is expected, with higher similarity between one’s behaviour in the collaborative condition and the partner’s in the complementary condition.

If, however, *proximal* space is only minimally modulated in collaborative contexts, then similarity with one’s own behaviour in the complementary condition should be higher than similarity with the partners’ behaviour in any condition.

**Figure 10:**
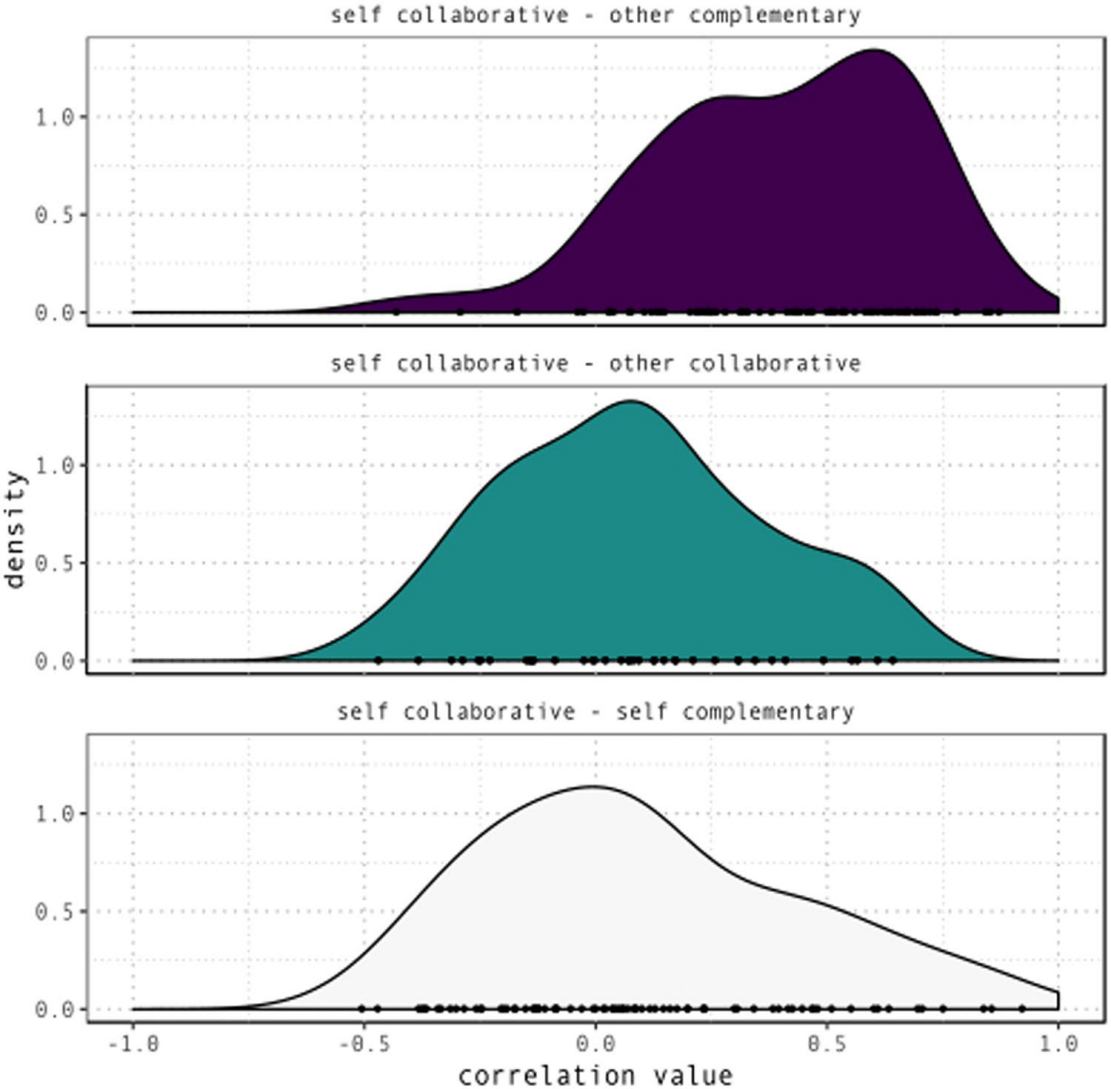
Density distributions of correlation between proportion of proximal demonstratives for each RelativeX-RelativeY value combination in the collaborative condition, and proportion of proximal demonstratives for their partner in the complementary condition (top), their partner in the collaborative condition (middle), themselves in the complementary condition (bottom). Higher correlation values denote higher similarity in coding of space as *proximal* vs. *distal* across participants and conditions.

To answer these questions, for each participant, condition and unique combination of values of RelativeX and RelativeY, we computed the proportion of trials where the outcome variable was a *proximal* demonstrative. This yielded, for each participant and condition, a map of empirical probabilities of targets being coded as *proximal* as a function of their sagittal and lateral distance from competing targets. For each participant, we then computed similarity (Pearson’s correlation) between this map in the collaborative condition and (a) the partner’s map in the complementary condition; (b) the partner’s map in the collaborative condition; (c) the participant’s own map in the complementary condition. Figure 10 displays the distribution of correlation values for each participant. Higher correlations denote larger overlap in participants’ coding of space.

As Fig. 10 shows, participants’ coding of space in the collaborative condition is overall most similar to their partner’s coding of space in the complementary condition. This pattern is statistically reliable. We fitted a mixed-effects linear regression with correlation values as outcome variable, and Correlation Type and Color (P1 and P2) and their interaction as fixed effects.

Correlation Type coded for whether a correlation value denoted similarity between one’s behaviour in the collaborative condition and either the partner’s behaviour in the complementary condition (*other complementary*), the partner’s behaviour in the collaborative condition (*other collaborative*), and one’s own behaviour in the complementary condition (*self complementary*).

The model included a random intercept for each pair and a random slope for the effect of correlation type. The model indicates that spatial coding in the collaborative condition was significantly more similar to the partner’s complementary condition than to the partner’s collaborative condition, β = −0.34, se = 0.06, t(73.35) = −5.35, p < 0.001, and with one’s own complementary condition, β = −0.38, se = 0.07, t(65.07) = = −5.42, p < 0.001. No other effects were significant. This lends support to the hypothesis that, in the collaborative condition, speakers remap their *proximal* space onto the partner’s action space. Details on the statistical model are reported in Supplementary Table S3.

## Discussion

Over two large-sample studies, we investigated how coding of spatial locations as functionally proximal vs. distal depends on the interaction between affordances for individual and joint action.

Using demonstrative reference as a proxy of near/far coding of space, we observed an overall prominence of spatial encoding strategies oriented to manual action in individual contexts or social contexts involving no collaboration. Objects were identified as *proximal* not only when located closer to participants on the sagittal axis but also when closer to the pointing hand on the lateral axis. In line with previous literature, we interpret this bias as a cognitive optimization strategy, responding to the need of minimizing sensorimotor transformations to perform manual action^13^.

The link between proximal/distal encoding of space and manual actions finds resonance in existing experimental literature on linguistic^28,35,36^ and non-linguistic^6,37^ spatial encoding. However, a major limitation of existing work on near/far spatial coding consists in its reliance on experimental paradigms where behavior is probed in single-participant settings. Crucially, these settings are not representative of a large portion of real-life experiences, where space is shared among individuals who are acting together, and where objects lend affordances for joint action.

In our studies, we investigated the *near/far* space distinction in both single- and multi-participant settings mirroring the structure of everyday interactions. We showed that the influence of manual affordances on space encoding observed in individual contexts is radically modulated by social factors. When participants label spatial locations as *proximal* or *distal* in collaborative contexts, their *proximal* space tends to shift towards the partner’s, a strategy which might facilitate the addressee’s task of disambiguating the intended locations^38^ and therefore smooth action performance. These partner-oriented adaptations are in line with characteristics of social behavior observed in non-linguistic joint action, where individuals adapt their behavior to minimize joint effort and optimally achieve shared goals^39^. For the individual agent, such flexible modulations of spatial representations might be cognitively more costly than sticking to a fixed coding scheme. However, they might be advantageous for the dyad: partner-oriented spatial encoding strategies arguably facilitate the partner’s task, and therefore improve the outcome of the joint action. Such phenomena would tightly parallel what observed in the context of motor interactions, where participants deviate from their optimal movement trajectory to ease their partner’s task of interpreting their motor intention^40,41^. However, further research is needed to test whether this is indeed the case by quantifying the impact of these modulations on the accuracy in joint task performance.

Modulations of peripersonal space triggered by engagement in social interaction have been previously reported in the literature. Teneggi and colleagues probed peripersonal space boundaries before and after engagement in an economic game, and found that they are shrunk or expanded as a function of the partner’s attitude in the game^14^. Similarly, peripersonal space boundaries interact with the social perception of others’ behavior and shared sensory experiences^15,42^.

Our results are compatible with these findings and extend them both in scope and methodology. In our paradigm, we investigated social modulations of spatial representations online, *during* task performance, focusing on how such adaptations mediate collaboration. As we show in our study, the effects of social adaptations can vary across tasks, ranging from subtle modulations of peripersonal space to an entire remapping into partner-centered coordinates. In interactions involving a symmetrical alternation of roles, egocentric manual biases are fully overridden by social ones, i.e. *proximal* space is entirely remapped onto the partner’s action space.

Participants thus modulate their spatial representations in a markedly *context-dependent* fashion, i.e. flexibly optimizing them for social action^15^. On the one hand, very asymmetrical modulation patterns might be optimal in cases where individual parts of the social task are independent, or where participants’ contribution to the joint task are unbalanced, congruent with asymmetrical motor coordination strategies observed in non-linguistic joint tasks^43^. On the other hand, a more symmetrical form of convergence towards a single shared model of space, as hypothesized by Peeters and colleagues^44,45^ might be advantageous for tasks building on bidirectional sharing of information along different turns, as in mutual adaptations characterizing alignment in linguistic interaction^17,46^. Alongside task-related constraints, individual differences in the ability or tendency to take others’ perspective could be hypothesized to act as a source of systematic variation in the amount of partner-oriented adaptations observed in spatial encoding^47^. Further research is needed to investigate in detail the relationship between information asymmetries and context-specific social adaptations in spatial reference, as well as the potential influence of individual traits on the use of flexible spatial encoding strategies during social interactions.

## Conclusion

Over two experiments, we investigated how functional organization of space into near and far locations is modulated by affordances for manual action and social interaction. We found that, in individual contexts, proximal space was centered on the hand, arguably optimizing representations for manual action. Manual biases were overridden by social factors in collaborative contexts, where proximal space was fully remapped onto the partner’s action space in interactions involving turn-taking. Action-oriented biases and social modulations interact in shaping spatial representations, a flexible adaptation strategy potentially facilitating joint task performance. This flexible coding of space provides humans with a powerful tool to efficiently adapt to a virtually unlimited range of social contexts, a unique and highly advantageous feature of human social behavior.

## Supplementary information

Supplementary Material

## Data Availability

Data and code are publicly available on the Open Science Framework at https://osf.io/qjxg9/.
