# Supplementary material for: This is for you: Social modulations of proximal vs. distal space in collaborative interaction
Source: Sci Rep. 2019 Oct 18;9:14967. doi: 10.1038/s41598-019-51134-8 (PMC6802403; doi:10.1038/s41598-019-51134-8)
Supplement: Supplementary file 1 — Supplementary Material [file 41598_2019_51134_MOESM1_ESM.docx]

***This* is for you:**

**Social modulations of proximal vs. distal space in collaborative interaction**

Roberta Rocca^1,2,*^, Mikkel Wallentin^1,2,3^, Cordula Vesper^1,2^, Kristian Tylén^1,2^

^1^ Department of Linguistics, Cognitive Science and Semiotics, Aarhus University, Denmark

^2^ Interacting Minds Centre, Aarhus University, Denmark

^3^ Center of Functionally Integrative Neuroscience, Aarhus University, Denmark

^*^ Corresponding author: [roberta.rocca@cc.au.dk](mailto:roberta.rocca@cc.au.dk) (RR)

# Supplementary Material

**Supplementary Table S1.** Overview of statistical model for Experiment 1.

|  | **Beta** | **SE** | **z** | **95%CI**  **lower** | **95%CI**  **upper** | **Odds**  **Ratio** | **p** |
| --- | --- | --- | --- | --- | --- | --- | --- |
| (Intercept) | 0,01 | 0,01 | 0,47 | -0,02 | 0,04 | 1,01 | 0,641 |
| RelativeY | -2,59 | 0,27 | -9,69 | -3,11 | -2,06 | 0,08 | <0.001^***^ |
| RelativeX | 0,32 | 0,02 | 16,78 | 0,28 | 0,35 | 1,37 | <0.001^***^ |
| RelativeY x RelativeX | 0,04 | 0,06 | 0,67 | -0,08 | 0,16 | 1,04 | 0,503 |
| RelativeY x Condition1  (baseline vs. other) | -0,07 | 0,03 | -2,51 | -0,12 | -0,02 | 0,93 | 0,012^*^ |
| RelativeY x Condition2  (complementary vs. collaborative) | -0,11 | 0,05 | -2,45 | -0,21 | -0,02 | 0,89 | 0,014^*^ |
| RelativeX x Condition1  (baseline vs. other) | 0 | 0,01 | -0,08 | -0,03 | 0,03 | 1 | 0,936 |
| RelativeX x Condition2  (complementary vs. collaborative) | 0,05 | 0,02 | 2,17 | 0 | 0,1 | 1,05 | 0,03^*^ |
| RelativeY x RelativeX x Condition1  (baseline vs. other) | 0 | 0,04 | -0,02 | -0,08 | 0,08 | 1 | 0,987 |
| RelativeY x RelativeX x Condition2  (complementary vs. collaborative) | -0,02 | 0,07 | -0,31 | -0,16 | 0,12 | 0,98 | 0,755 |

**Supplementary Table S2.** Overview of estimates from the statistical model for Experiment 2.

|  | **Beta** | **SE** | **z** | **95%CI**  **lower** | **95%CI**  **upper** | **Odds**  **Ratio** | **p** |
| --- | --- | --- | --- | --- | --- | --- | --- |
| (Intercept) | 0,01 | 0,04 | 0,22 | -0,07 | 0,09 | 1,01 | 0,824 |
| Color | 0,01 | 0,06 | 0,1 | -0,11 | 0,12 | 1,01 | 0,922 |
| RelativeX | -0,94 | 0,12 | -8,16 | -1,17 | -0,72 | 0,39 | <0.001^***^ |
| RelativeY | -2,21 | 0,23 | -9,69 | -2,66 | -1,76 | 0,11 | <0.001^***^ |
| Condition | 0,05 | 0,06 | 0,89 | -0,06 | 0,17 | 1,05 | 0,374 |
| Color x RelativeX | 1,16 | 0,16 | 7,27 | 0,85 | 1,47 | 3,19 | <0.001^***^ |
| Color x RelativeY | -0,4 | 0,18 | -2,26 | -0,74 | -0,05 | 0,67 | 0,024^*^ |
| RelativeX x RelativeY | -0,1 | 0,35 | -0,3 | -0,8 | 0,59 | 0,9 | 0,767 |
| Color x Condition | -0,04 | 0,08 | -0,49 | -0,2 | 0,12 | 0,96 | 0,624 |
| RelativeX x Condition | -1,8 | 0,17 | -10,43 | -2,13 | -1,46 | 0,17 | <0.001^***^ |
| RelativeY x Condition | 1,43 | 0,17 | 8,36 | 1,1 | 1,77 | 4,19 | <0.001^***^ |
| Color x RelativeX x RelativeY | 0,43 | 0,51 | 0,85 | -0,56 | 1,42 | 1,54 | 0,396 |
| Color x RelativeX x Condition | 4,83 | 0,25 | 19,47 | 4,34 | 5,31 | 124,83 | <0.001^***^ |
| Color x RelativeY x Condition | 1,05 | 0,24 | 4,29 | 0,57 | 1,52 | 2,85 | <0.001^***^ |
| RelativeX x RelativeY x Condition | 0,1 | 0,5 | 0,2 | -0,89 | 1,09 | 1,11 | 0,841 |
| Color x RelativeX x RelativeY x Condition | 0,27 | 0,74 | 0,37 | -1,17 | 1,72 | 1,31 | 0,712 |

**Supplementary Table S3.** Estimates from the statistical model for the exploratory correlation analysis.

|  | **Beta** | **SE** | **df** | **t** | **p** |
| --- | --- | --- | --- | --- | --- |
| (Intercept) | 0,42 | 0,04 | 77,28 | 9,74 | <0.001^***^ |
| CorrelationType  (other complementary vs other collaborative) | -0,34 | 0,06 | 73,35 | -5,35 | <0.001^***^ |
| CorrelationType  (other complementary vs self complementary) | -0,38 | 0,07 | 65,07 | -5,42 | <0.001^***^ |
| Color | -0,04 | 0,05 | 114 | -0,74 | 0,463 |
| CorrelationType x Color  (other complementary vs other collaborative) | 0,04 | 0,07 | 114 | 0,52 | 0,604 |
| CorrelationType x Color  (other complementary vs self complementary) | 0,15 | 0,07 | 114 | 2,11 | 0,037^*^ |
